# A novel technique to treat acquired Chiari I malformation after supratentorial shunting

**DOI:** 10.1007/s00381-016-3138-7

**Published:** 2016-06-11

**Authors:** Adriaan R. E. Potgieser, Eelco W. Hoving

**Affiliations:** Department of Neurosurgery, University Medical Center Groningen, Hanzeplein 1, P.O. Box 30.001, 9700 RB Groningen, The Netherlands

**Keywords:** Acquired Chiari I malformation, Ventriculoperitoneal shunt, Cranial vault thickening

## Abstract

**Purpose:**

The acquired Chiari I malformation with abnormal cranial vault thickening is a rare late complication of supratentorial shunting. It poses a difficult clinical problem, and there is debate about the optimal surgical strategy. Some authors advocate supratentorial skull enlarging procedures while others prefer a normal Chiari decompression consisting of a suboccipital craniectomy, with or without C1 laminectomy and dural patch grafting.

**Methods:**

We illustrate three cases of symptomatic acquired Chiari I malformation due to inward cranial vault thickening.

**Results:**

We describe a new surgical approach that appears to be effective in these patients. This approach includes the standard Chiari decompression combined with posterior fossa augmentation by thinning the occipital planum.

**Conclusion:**

Internal volume re-expansion of the posterior fossa by thinning the occipital planum appears to be an effective novel surgical strategy in conjunction with the standard surgical therapy of Chiari decompression.

## Introduction

The Chiari I malformation, or primary cerebellar ectopia, is one of the mildest forms of hindbrain herniation and is characterized by a downward displacement of the cerebellar tonsils through the foramen magnum into the cervical canal. The acquired Chiari I malformation is in fact a iatrogenic form of hindbrain herniation, first described after lumboperitoneal shunting [2, 4, 8, 17, 21], but also after multiple lumbar punctures [18], after baclofen pump placement [19], and it was later recognized that an acquired Chiari I malformation can also occur after cystoperitoneal and ventriculoperitoneal shunting [1, 3, 4, 6, 7, 9–12, 14, 20].

In a previous case series, 17 of 1700 patients treated with ventriculoperitoneal shunting developed a Chiari I malformation as a late complication, of which only five patients were symptomatic [1]. It is therefore a rare complication of supratentorial shunting, but it poses a difficult problem. In many cases, the only symptom is headache, which is also a common problem in the general population. In these patients, it may point at overdrainage, slit ventricle syndrome, hydrocephalus, or acquired Chiari I malformation. There are some similarities between the described cases, but the causes of the Chiari I development seem to differ because there is a difference in timing of symptoms. Furthermore, there has been debate about the optimal surgical strategy to alleviate symptoms. We present three illustrative cases of an acquired Chiari I malformation due to cranial vault thickening as a perceived late complication of supratentorial shunting and introduce a novel surgical strategy.

## Case reports

**Fig. 1:**
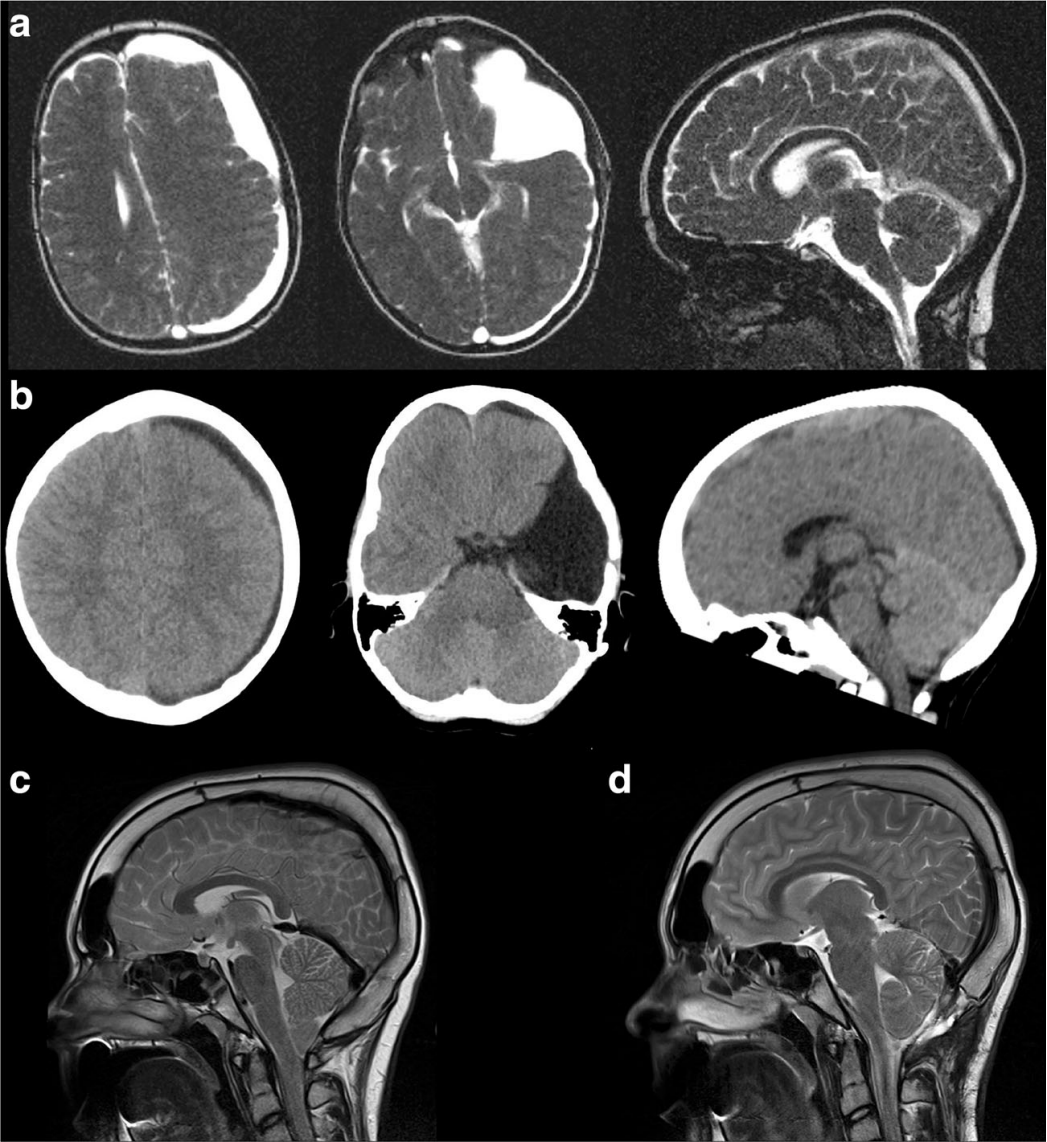
CT and MRI scans of a 3-year-old boy with a bleeding in a left frontotemporal arachnoid cyst, treated with a craniotomy with fenestration and marsupialization of the cyst. A large left-sided subdural hygroma developed at the location of the former arachnoid cyst (**a**) after which a subduroperitoneal drain was placed with good result (**b**). At the age of 14 years, he developed an acquired Chiari I malformation due to cranial vault thickening (**c**), which was treated with a standardized C0 augmentation, C1 laminectomy, and a dural patch, followed by thinning of the occipital planum. The postoperative MRI shows an adequate decompression with enlargement of the posterior fossa (**d**)

Case 1 is a 3-year-old boy with a bleeding in a left frontotemporal arachnoid cyst. He was treated with a craniotomy with fenestration and marsupialization of the arachnoid cyst. Half a year later, a subduroperitoneal drain without valve was implanted because of a persistent headache due to a large subdural hygroma at the location of the former arachnoid cyst (Fig. 1a). During the following years, the patient developed variable postural headaches attributed to liquor hypotension. Removing the drain led to immediate severe headache, and we decided to implant a new subduroperitoneal shunt with a PS medical medium valve. Subsequent shunt revisions over the years were performed with changing the valve to a Delta II, back to PS medical medium and finally to a Miethke 9/29. The latter valve resulted in an acceptable clinical condition (Fig. 1b) with symptoms of occasional mild headaches during the following years. At the age of 14, he developed a different kind of headaches, characterized by short attacks of headaches relieved by vomiting. An MRI scan showed an acquired Chiari I malformation due to thickening of the cranial vault with a subsequent decrease of intracranial volume especially within the posterior fossa, without signs of syringomyelia or spinal CSF leakage (Fig. 1c). We decided to augment the volume of the posterior fossa in combination with decompression of the Chiari without creating a risk of a descending cerebellum. Therefore, we modeled the thickened occipital planum to a normal size and subsequently placed back the thinned bone. During the same procedure, a standardized C0 augmentation, C1 laminectomy, and a dural patch were given. The patient has remained without symptoms now for more than a year. A control MRI showed an adequate decompression (Fig. 1d). The volume of the posterior fossa was estimated on the T2-weighted scans using Brainlab iPlan 3.0 Cranial. The tentorium, occiput, McRae line, and clivus served as boundaries of the posterior fossa. There was an increase in the size of the posterior fossa (188 cm^3^ preoperatively, 205 cm^3^ postoperatively).

**Fig. 2:**
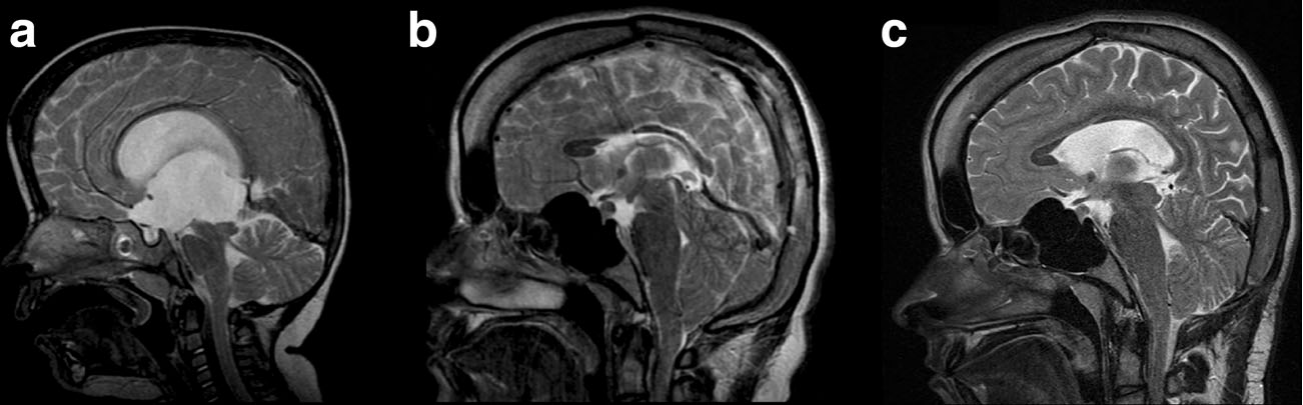
MRI scans of a 3-year-old girl with hydrocephalus due to aqueductal stenosis (**a**). She was treated with a third ventriculostomy and a ventriculoperitoneal shunt. Several years later, she developed an acquired Chiari I malformation with cranial vault thickening (**b**). After augmentation of the posterior fossa by thinning the occipital planum and decompression of C0 and a C1 laminectomy with dural patch grafting, the postoperative MRI scan shows an adequate decompression (**c**)

Case 2 is a 3-year-old girl with an increased intracranial pressure due to an aqueductal stenosis with hydrocephalus (Fig. 2a). We conducted a third ventriculostomy, which was complicated by a meningitis, external ventricular drainage, and eventually ventriculoperitoneal shunting (PS medical medium valve) with good result. Two years later, she developed persistent non-postural headaches. We decided to explore the ventriculostomy, which proved to be patent, and the ventriculoperitoneal shunt also functioned. After a period with mild headaches, she developed persistent headaches and a mild coordination disorder, which led to referral back to our clinic. A new MRI showed an acquired Chiari I malformation with clear cranial vault thickening (Fig. 2b) and a subsequent decrease in volume of the posterior fossa. A similar treatment strategy was chosen with augmentation of the posterior fossa by thinning the occipital planum and decompression of C0 and a C1 laminectomy with dural patch grafting. We also decided to upgrade the valve to a gravitational Miethke 10/30. The posterior fossa volume increased (from 155 cm^3^ preoperatively to 174 cm^3^ postoperatively). The patient’s headaches have diminished ever since. MRI showed an adequate decompression with a subtle increase in the size of the lateral ventricles (Fig. 2c).

**Fig. 3:**
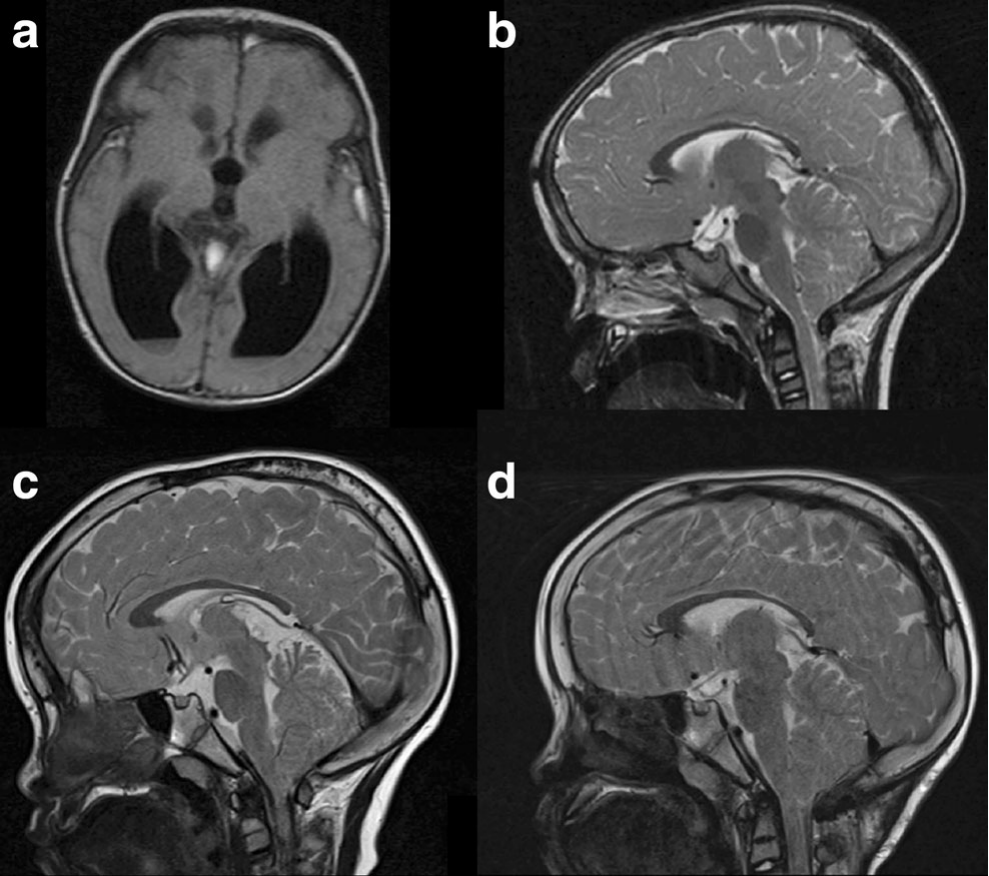
Case 3. MRI scan of a bleeding from a cyst in the right thalamus (**a**), treated with fenestration of the cyst, external ventricular drainage, and eventually a ventriculoperitoneal shunt. During follow-up, an asymptomatic Chiari I malformation was seen (**b**), that became symptomatic after a clear thickening of the cranial vault in the following years (**c**). The postoperative MRI scan shows an adequate decompression after a C1 laminectomy and posterior fossa decompression, during which we placed back the thinned occipital plane after remodeling it to a normal size (**d**)

Case 3 is a boy with X-linked recessive chondrodysplasia punctata (mutation in the CDPX1 gene), which is a very rare disease characterized by skeletal dysplasias [5]. Although not very well known, types of chondrodysplasia punctata can affect the craniocervical junction [14]. At the age of 1 month, he presented with a raised intracranial pressure due to a bleeding in a cyst in the right thalamus (Fig. 3a). We performed a third ventriculostomy with fenestration of the cyst and left an external ventricular drain. Two weeks after removal of the drain, the patient developed a raised intracranial pressure, and a ventriculoperitoneal shunt with PS medical low valve was inserted. During the following years, he had intermittent periods of non-postural headaches. A Chiari I malformation was diagnosed, but this was considered an asymptomatic finding (Fig. 3b). At the age of 6 years, the valve was upgraded and replaced by a Miethke 9/24. Half a year later, the boy had short paroxysmal non-postural headaches, and the MRI scan showed thickening of the cranial vault with an “acquired” Chiari I malformation (Fig. 3c). We performed a similar procedure as the previous two cases. A C1 laminectomy and posterior fossa decompression were performed in combination with thinning of the occipital planum (Fig. 3d). After this surgical procedure, the patient was immediately relieved from his symptoms. The posterior fossa volume increased (from 114 cm^3^ preoperatively to 127 cm^3^ postoperatively).

## Discussion

There has been a continuing debate about the optimal surgical treatment of the acquired Chiari I malformation. Some authors prefer supratentorial skull enlarging procedures [1, 4, 11, 12], which can be considered a major surgical procedure with potentially more morbidity than our approach. Others advocate a suboccipital craniectomy, with or without C1 laminectomy and dural patch grafting [9, 13], which can be considered as a standard procedure for the “normal” Chiari I malformation. In our cases, the Chiari I malformation coincides with acquired thickening of the skull with a subsequent decrease in volume of the posterior fossa. Our novel surgical approach to the acquired Chiari I malformation with inward skull thickening and subsequent loss of posterior fossa volume includes a standard Chiari decompression (C0/C1 decompression and dural patch grafting) combined with internal posterior fossa volume augmentation by thinning the occipital planum. In order to improve conditions for proper shunting, we tried to augment the size of the ventricles by inserting gravitational shunts in combination with the local treatment of the Chiari and posterior fossa as described. The combined surgical procedure of Chiari decompression and internal volume expansion of the posterior fossa by thinning of the occipital planum appears to be effective in our three cases, without creating new problems of a symptomatic descending cerebellum.

Based on the literature, three mechanisms of an acquired Chiari I malformation can be differentiated due to a difference in timing of symptoms. For both supratentorial shunting and lumbar shunting, early-onset cases (roughly within a year of placement) without cranial vault thickening have been described [6, 10, 17–20], while it is also seen as a late complication as was also the case in our patients [1, 3, 4, 12, 13].

An early-onset acquired Chiari I after lumboperitoneal shunting appeals to the pressure gradient theory [21]. A craniospinal pressure gradient is generated which basically sucks the cerebellum into the cervical canal. The successful treatment of an acquired Chiari I malformation caused by lumboperitoneal shunting by converting it to a ventriculoperitoneal shunt supports this theory [4, 17]. An early-onset acquired Chiari I after supratentorial shunting must have a different mechanism because no downward pressure gradient is generated. This may be caused by overdrainage of the ventricles, which leads to sagging of the brain as a whole, possibly in combination with a small (but previously sufficiently sized) posterior fossa. Our three cases had a late-onset development of the Chiari I malformation, characterized by modifications of the skull induced by the shunt. Initially named cephalocranial disproportion [7], Osuagwu et al. called it posterior cranial fossa disproportion, because they showed that there are normal cerebellar and supratentorial volumes in shunted children but a smaller posterior cranial fossa [16]. They propose that the etiology is an arrested posterior cranial fossa growth, similar to the etiology in the “normal” adult Chiari I malformation [15, 16]. However, they do not describe an abnormally thickened and inward growing cranial vault, as in our patients and those of many others [1]. It is possibly the combination of an arrested skull growth with subsequent inward cranial vault thickening due to chronic overdrainage that gives rise to this kind of acquired Chiari I malformation [1]. This seems evident in our cases, and our surgical approach appeals to this problem.

## Conclusion

We illustrate three cases of symptomatic acquired Chiari I malformation with cranial vault thickening as a perceived late complication of supratentorial shunting. Internal volume re-expansion of the posterior fossa by thinning the occipital planum appears to be an effective novel surgical strategy in conjunction with the standard surgical therapy of Chiari decompression.
